# Island-wide diversity in single nucleotide polymorphisms of the *Plasmodium vivax *dihydrofolate reductase and dihydropteroate synthetase genes in Sri Lanka

**DOI:** 10.1186/1475-2875-6-28

**Published:** 2007-03-09

**Authors:** Mette L Schousboe, Rupika S Rajakaruna, Ali Salanti, Hapuarachchige C Hapuarachchi, Gawrie NL Galappaththy, Ib C Bygbjerg, Priyanie H Amerasinghe, Flemming Konradsen, Michael Alifrangis

**Affiliations:** 1 Centre for Medical Parasitology, Institute for International Health, Immunology and Microbiology, Øster Farimagsgade 5, 1014 Copenhagen K, Denmark; 2 Department of Zoology, University of Peradeniya, Peradeniya 20400, Sri Lanka; 3 Department of Parasitology, Faculty of Medicine, P.O. Box 6, Thallagolla Road, Ragama, Sri Lanka; 4 Ministry of Health, Anti-Malarial Campaign Head Office, Colombo, Sri Lanka; 5 International Water Management Institute, Hyderabad, Andhra Pradesh, India

## Abstract

**Background:**

Single nucleotide polymorphisms (SNPs) in the *Plasmodium vivax *dihydrofolate reductase (*Pfdhfr*) and dihydropteroate synthetase (*Pvdhps*) genes cause parasite resistance to the antifolate drug combination, sulphadoxine/pyrimethamine (SP). Monitoring these SNPs provide insights into the level of drug pressure caused by SP use and presumably other antifolate drugs. In Sri Lanka, chloroquine (CQ) with primaquine (PQ) and SP with PQ is used as first and second line treatment, respectively, against uncomplicated *Plasmodium falciparum *and/or *P. vivax *infections. CQ/PQ is still efficacious against *P. vivax *infections, thus SP is rarely used and it is assumed that the prevalence of SNPs related to *P. vivax *SP resistance is low. However, this has not been assessed in Sri Lanka as in most other parts of Asia. This study describes the prevalence and distribution of SNPs related to *P. vivax *SP resistance across Sri Lanka.

**Subjects and methods:**

*P. vivax-*positive samples were collected from subjects presenting at government health facilities across nine of the major malaria endemic districts on the island. The samples were analysed for SNPs/haplotypes at codon 57, 58, 61 and 117 of the *Pvdhfr *gene and 383, 553 and 585 of the *Pvdhps *gene by applying PCR followed by a hybridization step using sequence specific oligonucleotide probes (SSOPs) in an ELISA format.

**Results:**

In the study period, the government of Sri Lanka recorded 2,149 *P. vivax *cases from the nine districts out of which, 454 (21.1%) blood samples were obtained. *Pvdhfr *haplotypes could be constructed for 373 of these. The FSTS wild-haplotype was represented in 257 samples (68.9%), the double mutant LRTS haplotype was the most frequently observed mutant (24.4%) while the triple mutation (LRTN) was only identified once. Except for two samples of the single mutated *Pvdhps *GAV haplotype, the remaining samples were wildtype. Geographical differences were apparent, notably a significantly higher frequency of mutant *Pvdhfr *haplotypes was observed in the Northern districts.

**Conclusion:**

Since SP is rarely used in Sri Lanka, the high frequency and diversity of *Pvdhfr *mutations was unexpected indicating the emergence of drug resistant parasites despite a low level of SP drug pressure.

## Background

*Plasmodium vivax *is the most geographically widespread of the four *Plasmodium *species infective to humans found throughout South and Central America, Asia, the Middle East, and parts of Africa and infects an estimated 70–80 million people annually [1]. Chloroquine (CQ)-resistant *Plasmodium falciparum*, and to a lesser extent CQ resistant *P. vivax*, is almost as endemic as malaria itself and alternatives such as the drug combination sulphadoxine/pyrimethamine (SP) have replaced CQ. Resistance to SP has recently emerged for *P. falciparum*, while for *P. vivax *it has been observed sporadically [2]. The molecular mechanisms involved in the development of SP resistance of the two species are most likely similar [3,4]. In *P. falciparum*, single nucleotide polymorphisms (SNPs) in codon (c) 51, c59 and c108 of the *Pfdhfr *gene and in c437 and c540 of *Pfdhps *gene provide pyrimethamine and sulphadoxine resistance, respectively and these SNPs combined result in high risk of SP treatment failure in vivo [5]. For *P. vivax*, the picture is more complex because pyrimethamine resistance possibly involve several SNPs [6]. However, some evidence support that resistance is mainly associated with mutations at c58 (S58R, occurring as two SNPs, either AGA (R_1_) or AGG (R_2_)) and c117 (S117N or S117T) with additional mutations at c57 (F57L-existing as three SNPs, CTC (L_1_), TTG (L_2_) and TTA (L_3_)) and c61 (T61M) in the *Pvdhfr *gene [3,4,6-8]. The quadruple mutant haplotype (57L+58R+61M+117T) has been shown to correlate with SP treatment failure in vivo [8] and increases *P. vivax *resistance to pyrimethamine by more than 500 times [4,6].

Presumably, *P. vivax *sulphadoxine resistance is caused by SNPs in the *Pvdhps *gene. Based on homology models of both *P. falciparum *and *P. vivax *DHPS enzymes, Korsinczky *et al*. predicted that the *P. vivax *wildtype at c585 (V585) possibly cause some level of innate sulphadoxine resistance, while SNPs at c383 (A383G) and c553 (A553G) in *Pvdhps *most likely increase resistance levels [9]. Imwong *et al*. showed that only in regions with high SP usage, SNPs in both *Pvdhfr *and *Pfdhps *were observed and, furthermore, parasites harbouring multiple mutations in *Pvdhfr *and *Pvdhps *were cleared more slowly from the blood of patients following SP treatment [10]. Therefore, *P. vivax *SP resistance is most likely measurable by examining the frequency of SNPs in both the *Pvdhfr and Pvdhps *genes.

In Sri Lanka, CQ plus primaquine (PQ) and SP plus PQ are used as 1^st^- and 2^nd^-line treatment, respectively, against uncomplicated malaria infections, although CQ resistant *P. falciparum *infections have been reported since 1984 and *P. falciparum *SP resistance has been observed recently [11]. *P. vivax *resistance to either CQ or SP has not been recorded on the island. This study investigated the frequency of SNPs/haplotypes in the *Pvdhfr *(at c57, 58, 61 and 117) and *Pvdhps *(at c383, 553 and 585) genes in samples collected from nine districts with endemic *P. vivax *malaria in Sri Lanka over a 1 1/2-year period. The detection of SNPs/haplotypes in *Pvdhfr *and *Pvdhps *was performed by applying a new simple enzyme-linked immunosorbent assay (ELISA) using sequence specific oligonucleotide probes (SSOPs) similar to the method detecting SNPs/haplotypes in *P. falciparum dhfr*, *dhps *and *crt *[12].

## Materials and methods

The samples originated from individuals seeking treatment for malaria at government health facilities located in nine different malarious district across Sri Lanka. In Sri Lanka the great majority of individuals with perceived malaria seek treatment at government facilities [13]. Samples were collected by routine staff at the facilities trained by the Anti-Malaria Campaign (AMC) of Sri Lanka from September 2004 to March 2006, thereby including the traditionally malaria peak transmission seasons in January and one lower peak season around July. Finger prick blood from patients with single *P. vivax *or mixed *P. vivax*/*P. falciparum *infections, diagnosed by microscopy were spotted on filter paper and sealed in individual zip-lock bags. DNA extraction was carried out by the chelex-100 method as described in [12].

As positive controls of the various *Pvdhfr *SNPs/haplotypes, 8 *P. vivax dhfr *allele samples, kindly provided by Carol Sibley (Dept. of Genome Sciences, University of Washington) were used [4]. These represent each of the ten most common c57, 58, 61 and 117 *Pvdhfr *SNPs/haplotypes. Furthermore, one positive control consisting of a 50 bp DNA fragment was designed mimicking a specific mutated *Pvdhfr *sequence, comprising the L_3_-mutation in c57 (TTA) and R_2_-mutation in c58 (AGG) (PcL_3_R_2_T in table 1) biotinylated at the 5'-end by the supplier (MWG Biotech, Riskov, Denmark). Likewise for positive controls of *Pvdhps *338G and 553G, 50 bp DNA fragments were designed mimicking these specific SNPs (Pc383G and Pc553G in Table 1).

**Table 1:** The sequence specific oligonucleotide probes (SSOPs) targeting SNPs/haplotypes in c57, 58, 61 and 117 of the *Pvdhfr *gene and c383, 553 and 585 in the *Pvdhps *gene and artificial positive controls.

SSOP	SSOP sequence †	Washing temperature §	Incubation time ¶
*Pvdhfr*		(C°)	Min.
57/58/61 FST	AC **TTC AGC **TCG GTG **ACG **A	60	12
57/58/61 FR_1_T	AC **TTC AGA **TCG GTG **ACG **A	60	10
57/58/61 FR_2_T	AC **TTC AGG **TCG GTG **ACG **A	60	10
57/58/61 L_1_R_2_M	AC**CTC AGG **TCG GTG **ATG **A	62	10
57/58/61 L_2_ST	AC **TTG AGC **TCG GTG **ACG **A	60	10
57/58/61 L_2_R_1_T	AC **TTG AGA **TCG GTG**ACG **A	64	10
57/58/61 L_3_R_2_T	AC **TTA AGG **TCG GTG **ACG **A	62	10
117 S	G AGA AGC **AGC **TGG GAG AG	60	12
117 N	G AGA AGC **AAC **TGG GAG AG	60	10
117 T	G AGA AGC **ACC **TGG GAG AG	60	10
			
*Pvdhps*			
383A	A TCG TCC **GCC **CCT TAT GT	64	10
c383 G	A TCG TCC **GGC **CCT TAT GT	64	10
c553A	TC GGC CTG GGG TTT**GCC **A	64	10
c553G	TC GGC CTG GGG TTT**GGC **A	64	10
c585V	C TTT ATT **GTC **CAC TGC AT	64	10
			
Positive controls*			
*Pvdhfr*	Sequence		
PcL_3_R_2_T	TCCGTCGATATGAAGTAC**TTAAGG**TCGGTG**ACG**ACCTACGTGGATGAGTC		
			
*Pvdhps*			
Pc383G	GATTGACATCGGGGGGGAATCGTCC**GGC**CCTTATGTGGTCCCCAATCCGA		
Pc553G	CTTTGATGTCGGCCTGGGGTTT**GGC**AAAAAGCACGACCAGTCTATTAAGC		

† Sequences in **bold **indicate the codon in which the SNP occurs.

§ Optimal TMAC-washing temperature.

¶ Optimal incubation time for TMAC-wash

* Artificial positive controls mimicking specific mutated *Pvdhfr *or *Pvdhps *sequences.

The outer and nested *Pvdhfr *PCR protocols used are described in [14], with the exception that the reverse nested primer, KH-3R was biotinylated at the 5'-end by the supplier (MWG Biotech, Riskov, Denmark). The outer *Pvdhps *PCR primers used (PvDHPS-D and PvDHPS-B) and protocols are described by [9]. The nested *Pvdhps *primers were designed; NL-1 (5'-GCGAGCGTGATTGACATC-3') and NR-1-(5'-GCTCATCAGTCTGCACTCC-3') where the reverse primer, NR-1, was biotinylated at the 5'-end. The outer and nested *Pvdhps *PCR were performed as follows: denaturation at 94**°**C for 2 min followed by 40 cycles of 94**°**C for 30 sec, 50**°**C for 30 sec and 65°C for 1 1/2 min and subsequently a 5 min extension step at 65°C.

A SSOP-ELISA, similar to the method for SNP/haplotype analysis of *P. falciparum dhfr*/*dhps *was developed [12], however using pre-coated streptavidin plates (Nunc, Roskilde, Denmark). The 3'-end digoxigenin-conjugated SSOPs designed to target the most common *Pvdhfr *and *Pvdhps *SNP/haplotypes including the time and temperatures in the two rounds of high stringency washing with tetra-methyl-ammonium chloride (TMAC) is given in table 1. Scoring of ELISA data were performed as described elsewhere [12]. To verify the results, Restriction Fragment Length Polymorphism (RFLP) was performed on a subset of the samples using enzymes and methods described by [15]. Polymorphisms in c383 of the *Pvdhps *gene were identified by digestion with the restriction enzyme HaeIII (New England Biolabs, Medinova, Glostrup, Denmark).

Sequencing was performed on a subset of samples to clarify some of the *Pvdhfr *and *Pvdhps *haplotypes; PCR products with A-overhang were cloned into the TOPO TA vector according to manufacturing procedures (Invitrogen), and plasmids were prepared using MiniPrep spin columns (Omega Biotech). Sequencing was done on an ABI Prism 377 (Perkin-Elmer) using the Big Dye terminator reaction mix (Perkin-Elmer).

Ethical clearance for this project was granted by the Committee on Research and Ethical Review at the Faculty of Medicine, Peradeniya, Kandy and verbal consent was obtained from participants, parents and/or guardians.

## Results

In the study period, AMC recorded a total of 2717 *P. vivax *cases in the country, out of which, 2,149 cases came from the nine districts included in this study (79.1%). 454 (21.1%) blood samples from these districts, representing a large range of catchments efficiencies from 7.7% (Monaragala) to 67.5% (Polonnaruwa) were examined. The samples were analysed for SNPs/haplotypes at position c57, 58, 61 and 117 of the *Pvdhfr *gene and c383, 553 and 585 (only detection of the wildtype V585) of the *Pvdhps *gene using an array of SSOPs. Samples either repeatedly PCR negative or negative in one or more of the *Pvdhfr *or *Pvdhps *codons were omitted from the analysis.

**Figure 1:**
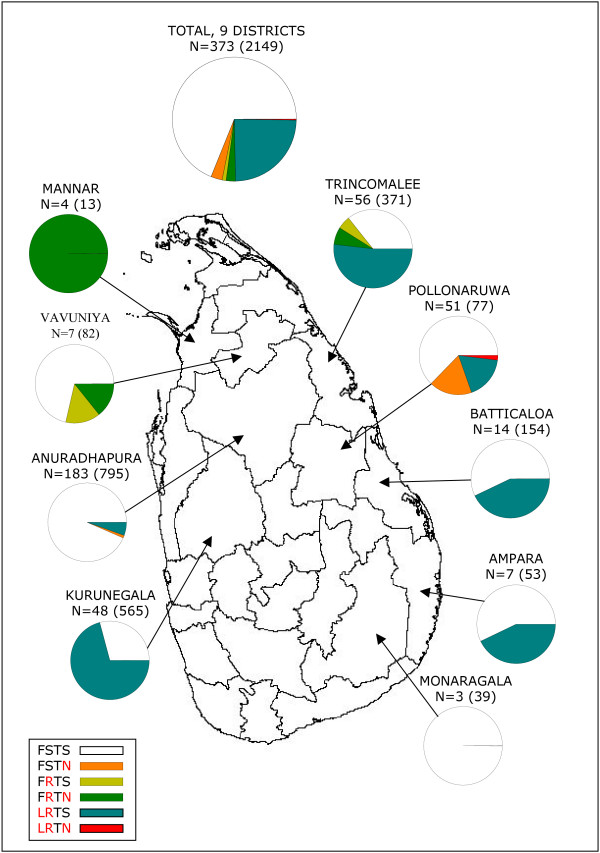
**The distribution of *Plasmodium vivax dhfr *haplotypes at c57 (F->L), 58 (S->R), 61 (T) and 117 (S->N) in samples collected from *P. vivax *positive patients in 9 districts of Sri Lanka**. The number in brackets is the actual number of *P. vivax *positive cases reported by Anti Malaria Campaign of Sri Lanka in the study period September 2004–March 2006. In red: the wild type to mutant type change in amino acids

For *Pvdhfr*, haplotypes could be constructed for 373 samples (84.6 %) including 25 samples with mixed haplotype infections, but where a major haplotype could be deduced (Figure 1). The *Pvdhfr *FSTS wild haplotype was represented in 257 samples (68.9 %) while the remaining samples comprised of five different mutant haplotypes at c57, 58 and 117 only: as F57L_3 _(TTC→TTA), S58R_2 _(AGC→AGG) and S117N. The double mutant LRTS haplotype was the most frequently mutated haplotype, observed in the districts Anuradhapura (frequency (f): 0.05, n = 10), Trincomalee (f: 0.52, n = 29), Pollonaruwa (f: 0.18, n = 9), Batticaloa (f: 0.43, n = 6), Kurunegala (f: 0.71, n = 34) and Ampara (f: 0.43, n = 3). The 117N mutation was less frequently observed (in total: f: 0.06, n = 21), as either the single mutated haplotype FSTN (only in Polonnaruwa and Anuradhapura), the double mutated haplotype, FRTN (only in the Northern districts of Mannar, Vavuniya and Trincomalee). The triple mutation (LRTN) was only identified once in the district Pollonaruwa and as a mixture of LRTN/LRTS with the LRTN in majority. A significantly higher frequency of mutant haplotypes (FSTN, FRTN and LRTS) was observed in the Northern districts (Mannar, Trincomalee and Vavuniya) compared to the other districts (χ^2 ^= 36.3, P ≤ 0.001). There was a general tendency for an increase in the frequency of mutant haplotypes late in the study period. However, due to a skewed temporal collection of samples from the various districts the differences was not analysed further.

A subset of samples analysed by sequencing (mainly to confirm the c57L_3_) and by digestion of c58 and 117 in the *Pvdhfr *gene by RFLP confirmed the data obtained be the *Pvdhfr *SSOP-ELISA.

The *Pvdhps *haplotypes could be constructed for 368 of the 373 *Pvdhfr *positive samples (98.7 %). Wildtype haplotypes at c383, 553 and 585 (AAV) was seen in 366 of these samples, while two samples from Trincomalee were of the single mutated GAV haplotype. These were confirmed by sequencing. Both samples expressed the double mutated FRTN haplotype in *Pvdhfr*.

## Discussion

The present descriptive study analysed sulphadoxine/pyrimethamine (SP) resistance-related SNPs in the *P. vivax dhfr *and *Pvdhps *genes in samples originating from nine districts in Sri Lanka, a country were both CQ and SP (in combination with primaquine) is still regarded as efficient treatment against uncomplicated *P. falciparum *and/or *P. vivax *infections. The analysis identified six different haplotypes of *Pvdhfr *while for *Pvdhps*, only wildtypes were identified except for two cases.

The double mutant haplotype LRTS (F57L, S58R, T61,117S) was the most frequent mutant haplotype and not as expected as a combination of S117N and S58R (FRTN) as observed previously [6,8,16] and in a recent study from India [17]. The *P. vivax *triple mutant haplotype LRTN, previously found in Thailand and shown to be associated with reduced ability of patients to decrease parasites ratios [15] was only found once and the quadruple LRMT mutant haplotype causing a high risk of SP treatment failures [8] was not detected, thus indicating that SP (with primaquine) is still efficient against *P. vivax *infections in Sri Lanka. Nevertheless, it is surprising that almost one third of the tested *P. vivax *infections were mutated in the *Pvdhfr *gene, despite that, officially, SP is only used as second-line drug against CQ treatment failures of *P. falciparum*. A recent study investigating the availability of SP in privately-owned drug vendor shops in Sri Lanka found that SP was virtually absent from the shops [18], thus the specific drug pressure is unlikely to be caused by unauthorized use. More plausible, the mutations are not only an indication of emerging pyrimethamine resistance, but instead reflect the overall antifolate pressure in Sri Lanka. Presently, antifolates such as dapsone, co-trimoxazole and trimethoprim are for instance used against urinary tract infections and chronic bronchitis on the island. Alternatively, similar to development of *P. falciparum *resistance to pyrimethamine *in vivo*, *P. vivax *populations are occasionally exposed to sub-therapeutic levels of pyrimethamine when re-infecting recently SP-treated patients thereby providing optimal conditions for the emergence of SP tolerant *P. vivax *parasites [19]. Thus, even low drug pressure may facilitate the emergence of drug tolerant/resistant parasites and this may particularly be the case for *P. vivax *that to a larger extend than *P. falciparum *possibly can persist in the host unnoticed.

The frequency of *Pvdhfr *mutant haplotypes was significantly higher in the most Northern regions (Mannar, Vavuniya and Trincomalee) than the rest of the districts examined. This might be indirectly caused by the civil unrest resulting in a shortage of trained medical personnel, non-accurate malaria diagnosis and an underestimation of malaria infections mainly in the Northern part of Sri Lanka [20]. Furthermore, the FRTN haplotype was only observed in the Northern districts and it may be speculated that human migration between Southern India and the Northern part of Sri Lanka has introduced this particular haplotype from the Southern district of Chennai where it is highly prevalent [17]. The limited number of samples received from some districts, e.g. Monaragala, Ampara, Batticaloa, Mannar and Vavuniya, limits interpretation.

The high frequency of mutant haplotypes related to pyrimethamine resistance is worrying because it indicates that drug tolerant/resistant *P. vivax *parasites have evolved despite a low level of SP drug pressure, possibly attributed to the use of other antifolate drugs. It is not known whether these mutant *P. vivax *haplotypes do exhibit SP resistance *in vivo*.

## Authors' contributions

RSR, GNLG, ICB, PHA, FK and MA designed the study, MLS carried out the molecular studies, MA, FK and MLS analysed the data, AS performed the sequencing experiments. HCH provided valuable inputs in the drafting of the manuscript. All authors read and approved the final manuscript.
